# Structure-based design of anticancer drugs based on β-elemene: Research foundations and development potential

**DOI:** 10.1016/j.jpha.2025.101325

**Published:** 2025-05-03

**Authors:** Haiyi Chen, Yuntao Yu, Chenghong Hu, Lehuang Zhou, Zhe Wang, Odin Zhang, Yi Wang, Tian Xie

**Affiliations:** a School of Pharmacy, Hangzhou Normal University, Hangzhou, 311121, China; b Key Laboratory of Elemene Class Anti-Cancer Chinese Medicines, Engineering Laboratory of Development and Application of Traditional Chinese Medicines, Collaborative Innovation Center of Traditional Chinese Medicines of Zhejiang Province, Hangzhou Normal University, Hangzhou, 311121, China; c College of Pharmaceutical Sciences, Zhejiang University, Hangzhou, 310058, China

**Keywords:** β-elemene, Drug design, Virtual screening, Molecular generation

## Abstract

β-elemene, a bioactive compound derived from traditional Chinese medicine (TCM), has been clinically used in cancer therapy. However, its molecular physicochemical properties require further optimization, and its precise anticancer mechanisms remain unknown. In modern drug development, structure-based drug design (SBDD) has significantly conserved resources, with computer-aided techniques such as molecular docking and molecule generation playing essential roles. A comprehensive review of existing molecular biology studies and virtual docking experiments led to the hypothesis that methyltransferase-like 3 (METTL3) may serve as a potential target of β-elemene. This discovery establishes a scientific foundation for integrating advanced, rational drug design strategies with β-elemene to enhance the therapeutic efficacy of TCM. Moreover, current (AI)-based molecular generation models were examined, focusing on *de novo* molecular generation and lead optimization models. Their applications in the rational drug design of β-elemene were preliminarily explored to identify potential strategies for developing more potent anticancer derivatives by analyzing ligand-receptor interactions.

## Highlights

• First systematic study of β-elemene’s anticancer mechanism via ligand-protein interactions.
• Docking reveals novel insights for β-elemene modifications, proposed for the first time in academia.
• AI-based molecular generation of β-elemene derivatives introduced, with preliminary examples provided.
• CADD-driven optimization strategy for β-elemene remains applicable beyond METTL3 for other anticancer targets.

## Graphical abstract

**Figure:**
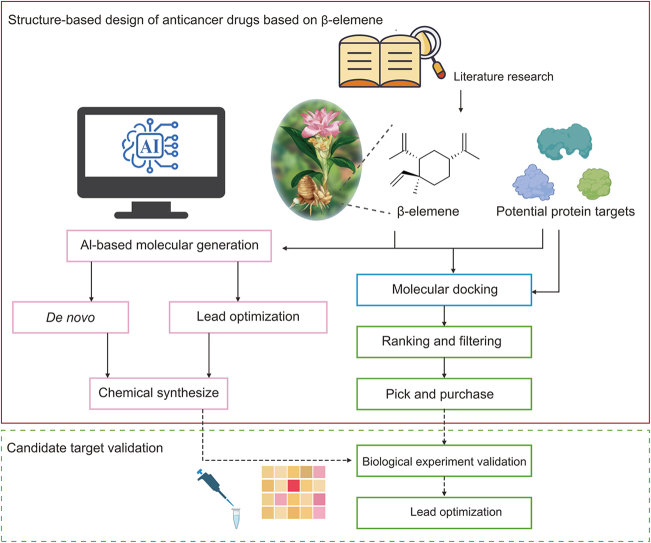

## 1 Introduction

Current cancer treatment primarily relies on surgery, radiotherapy, and chemotherapy to eliminate malignant cells. However, these conventional approaches often disrupt normal cellular function, resulting in severe adverse effects that significantly reduce quality of life. However, anticancer natural compounds, particularly those derived from traditional Chinese medicine (TCM), have attracted growing interest due to their favorable safety profiles, characterized by low toxicity and minimal side effects [1]. β-elemene, a prominent anticancer compound derived from TCM, is extracted and purified from the herb *Curcuma wen yujin*. This extract consists of multiple isomers, with β-elemene as the primary bioactive component [2,3]. Structurally, β-elemene is a lipid-soluble sesquiterpene with a molecular weight of 204.35 and the chemical name (1*S*,2*S*,4*R*)-1-ethenyl-1-methyl-2,4-*bis*(prop-1-*en*-2-yl)cyclohexane. Its anticancer properties were first identified in 1981, and in 1994, its injectable formulation received regulatory approval in China for clinical use as an antitumor agent [4]. To date, β-elemene remains the only known anticancer drug composed exclusively of carbon and hydrogen atoms, without aromatic rings [3].

**Fig. 1:**
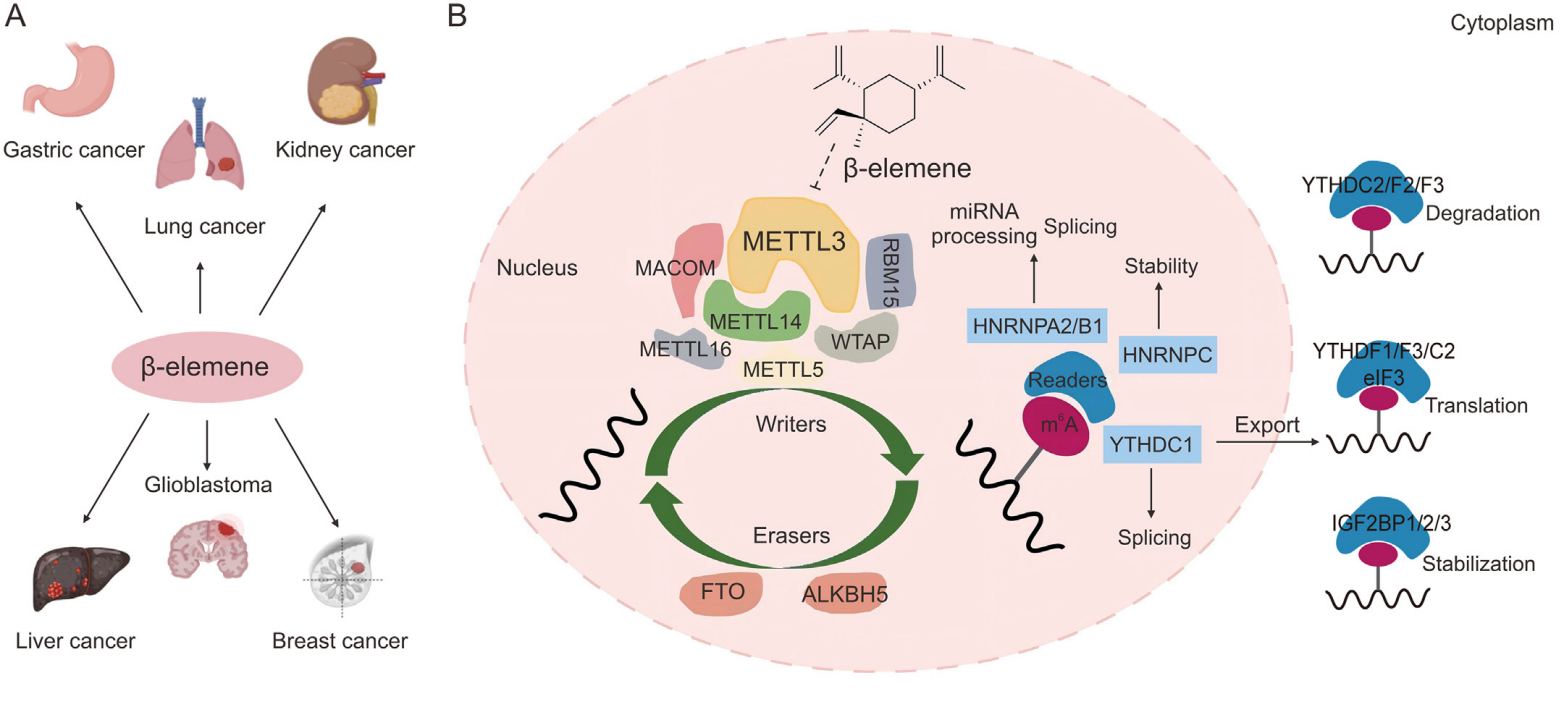
Therapeutic implications of β-elemene. (A) Anticancer effects of β-elemene. (B) Schematic representation of the *N*^6^-methyladenosine (m^6^A) modification process of messenger RNA (mRNA) that β-elemene may affect in eukaryotic cells. MACOM: m^6^A methyltransferase complex; METTL3: methyltransferase-like 3; RBM15: RNA binding motif protein 15; WTAP: wilms tumor 1-associated protein; FTO: fat mass and obesity-associated protein; ALKBH5: AlkB homolog 5; miRNA: microRNA; HNRNPA2/B1: heterogeneous nuclear ribonucleoprotein A2/B1; HNRNPC: heterogeneous nuclear ribonucleoprotein C; YTHDC1: YTH domain-containing protein 1; eIF3: eukaryotic translation initiation factor 3; IGF2BP1/2/3: Insulin-like growth factor 2 mRNA-binding protein 1/2/3. Created with BioRender.com.

Multiple studies have demonstrated that β-elemene showed therapeutic effects against various common cancers [3], such as gastric cancer [5,6], lung cancer [7,8], kidney cancer, liver cancer [9], glioblastoma [10] and breast cancer [11,12] (Fig. 1A), both *in vitro* and *in vivo*, as well as in clinical treatments [13]. Moreover, when combined with other anticancer chemotherapy agents, β-elemene has demonstrated the potential to overcome cancer cell resistance [14,15], improve the effectiveness of chemotherapy [16,17], and reduce treatment-related side effects [18,19]. While β-elemene may not exert direct cytotoxic effects on cancer cells, its ability to suppress cancer cell growth, promote apoptosis, and show minimal toxicity highlights its distinct advantage as a bioactive compound in TCM [20]. However, due to the unusual molecular structure feature of β-elemene, its clinical application has been limited by poor water solubility, molecular instability, and relatively weak antitumor activity [21]. To overcome this challenge, extensive research in pharmaceutics has been directed toward the development of advanced formulations designed to enhance the targeted delivery of β-elemene to cancer cells, thereby maximizing its therapeutic potential. Following the formulation guidelines of TCM [3], liposomal drug delivery systems have been developed for the treatment of various cancers, including bladder cancer and glioma [22,23]. Different multicenter studies have clinically proven the safety and efficacy of elemene liposome series across multiple cancer diseases [3]. Other pharmaceutical approaches, such as nano heterojunction-mediated thermoelectric strategy [24], stanene-based nanosheets with ultrasound [25], and arsenene nanodots [26], have also been used in the preparation of β-elemene anticancer therapies.

The production of β-elemene remains dependent on natural sources, as they are a key active component of TCM [27,28]. Despite advancements in pharmacological strategies that have somewhat improved their therapeutic application, the specific molecular targets of β-elemene remain unclear. Moreover, the precise molecular targets of β-elemene have yet to be fully elucidated, resulting in inefficiencies in drug utilization and substantial loss of bioactive compounds, posing a significant limitation. Advancements in structural determination techniques and the rapid progress of artificial intelligence (AI)-driven structural prediction algorithms have established structure-based drug design (SBDD) as a leading strategy in modern drug development [29]. This strategy substantially improves efficiency by optimizing resource consumption and reducing the time required for drug discovery. A comprehensive understanding of reference ligands and target protein binding sites enables the effective application of computer-aided drug design (CADD) techniques, including molecular docking-based virtual screening and AI-driven molecular generation, to develop lead compounds with high activity and innovative structures. This study elucidated the molecular mechanisms of β-elemene, identified its potential anticancer target proteins, and examined the feasibility of rational drug design strategies for further optimization.

## 2 Anticancer target protein of β-elemene

Previous studies have shown that in addition to its broad-spectrum anticancer activity [3], β-elemene disrupts critical cellular processes in cancer cells and impedes cell cycle progression [3,13]. However, the specific mechanisms underlying its anticancer effects have not been fully elucidated. In eukaryotic cells, biological activities are tightly regulated via gene transcription and expression, with one of the most prevalent modifications being the methylation of the *N*^6^ position of adenosine in messenger RNA (mRNA), referred to as *N*^6^-methyladenosine (m^6^A) [[30], [31], [32]]. This modification is highly involved in all stages of the RNA life cycle. Undoubtedly, m^6^A modification is crucial for the growth and development of mammals, and abnormalities in related proteins are directly reflected in individual phenotypes [33,34]. Extensive research has demonstrated a significant correlation between m^6^A modifications and cancer progression, with aberrant expression of m^6^A regulatory proteins directly impacting critical cellular processes such as proliferation, apoptosis, autophagy, and migration in cancer cells [35,36].

Recently, the association between the anticancer activity of β-elemene and m^6^A modifications has been demonstrated in lung cancer cells, marking a significant advancement in understanding the mechanisms of β-elemene in cancer treatment [7]. Based on the functionality, the proteins involved in m^6^A can be categorized into three, i.e., methyltransferases (writers), demethylases (erasers), and m^6^A-binding proteins (readers) [37] (Fig. 1B). During gene transcription and expression, writer proteins i.e., methyltransferase-like 3 (METTL3), METTL14, Wilms’ tumor 1-associating protein (WTAP), and other associated factors, form complexes that simultaneously bind to the substrate *S*-adenosylmethionine (SAM) and the target mRNA [[38], [39], [40]]. This interaction facilitates the transfer of a methyl group from SAM to the *N*^6^ position of adenine in mRNA. Moreover, eraser proteins such as fat mass and obesity-associated protein (FTO) and AlkB homolog 5 (ALKBH5) can dynamically reverse m^6^A modifications, thereby regulating RNA methylation [33,41]. After completing m^6^A modifications, specific reader proteins recognize and bind to the modified mRNAs, thereby facilitating essential cellular processes such as translation, RNA stabilization, degradation, and translocation [42]. Fig. 1B depicts the regulatory functions of these three classes of m^6^A-associated proteins in mRNA modulation. Theoretically, all three categories of m^6^A-related proteins can serve as targets for the design of small-molecule anti-cancer drugs. For example, in the case of writers, current studies have confirmed that the overexpression of the vital protein METTL3, which catalyzes m^6^A methylation, is closely associated with cancer development [43]. While WTAP, a structural assembly protein within the writer complex, does not directly catalyze m^6^A modifications, and it remains a potential therapeutic target for cancer [44]. Among the two major eraser proteins, ALKBH5 is a potential target for treating ovarian and gastric cancers [45,46], while FTO is associated with breast cancer treatment [47]. Among the various reader proteins, YT521-B homology domain-containing protein 2 (YTHDC2) has been involved in colon cancer metastasis and holds potential as a diagnostic biomarker and a therapeutic target for colon cancer [48]. Moreover, insulin-like growth factor 2 mRNA-binding protein (IGF2BP), which plays a crucial role in stabilizing mRNA, has been associated with cancer progression [49]. While extensive research has explored the anticancer mechanisms of β-elemene from the perspective of signaling pathway regulation, studies investigating receptor-ligand interactions at the microscopic level within the complex network of m^6^A-related target proteins remain limited. Current research suggests that the anticancer effects of β-elemene are mediated through the downregulation of METTL3 expression and its potential binding to METTL3 as an inhibitor [14,50]. Therefore, the subsequent discussion in this paper will primarily center on METTL3.

METTL3 is the most important protein in the writer complex that directly catalyzes m^6^A methylation. It forms a heterodimer with METTL14 via an intricate hydrogen bond network, creating a positively charged groove at the junction for mRNA binding [38]. METTL3 contains a prominent binding site for the catalytic substrate SAM, while no similar site has been identified on METTL14. Other writer proteins, including WTAP and Vir-like m^6^A methyltransferase-associated protein (VIRMA), function as auxiliary components that assemble with the METTL3-METTL14 heterodimer to form the m^6^A catalytic complex [[51], [52], [53]]. During catalysis, the methyl group from SAM is efficiently transferred by this complex to the *N*^6^ position of adenosine in mRNA, after which the by-product *S*-adenosyl homocysteine (SAH) dissociates from the binding site of METTL3, making way for the next SAM molecule for interaction. Considering the catalytic properties of the METTL3-METTL14 heterodimer, the design of inhibitors that specifically occupy the SAM binding site constitutes a promising strategy for anticancer drug development. In 2020, Liu et al. [14] indicated that β-elemene might lower m^6^A modification levels in gefitinib-resistant cancer cells by inhibiting METTL3 and provided a molecular docking analysis using AutoDock tools, demonstrating the binding complex of β-elemene with METTL3, where β-elemene occupied the SAM binding site. Moreover, Bedi et al. [54] and Li et al. [55] investigated the crystal structure of the sinefungin-METTL3 complex. They identified three distinct structural components of sinefungin: i) an aromatic ring that occupies the P_1_ region of the binding pocket, where SAM binds, ii) a rigid linker fragment, and iii) a hydrophobic segment extending into the P_3_ region of the binding pocket, featuring amino groups that interact with the DPPW motif. β-elemene, a hydrophobic molecule, shows structural compatibility with the P_2_ region, and several of its derivatives share molecular features similar to those of sinefungin [56]. Therefore, it hypothesizes that these derivatives may serve as potential ligands for METTL3. This aspect will be discussed in detail in the following sections.

In addition to m^6^A-related proteins, β-elemene may have other potential target proteins. However, current studies have yet to yield significant insights into these interactions from a receptor-ligand perspective. As an overview, several representative studies on alternative target proteins are summarized here, with the expectation that future research will further explore receptor-ligand interactions in more detail. Histone deacetylases (HDACs), a class of epigenetic enzymes, serve as targets for anticancer drugs, and several HDAC inhibitors have been used in clinical cancer treatment. However, these are limited to hematological malignancies and often lead to drug resistance. Gao et al. [57] synthesized a series of β-elemene derivatives based on the structure of the traditional HDAC inhibitor, vorinostat, among which the *N*-alkyl-*N*-hydroxyl carboximate compound 11i demonstrated anticancer activity in both *in vitro* and *in vivo* experiments. However, this study did not provide direct evidence of 11i binding to HDAC. After that, Gao et al. [57] synthesized derivatives 27f and 39f using β-elemene as a core scaffold, which showed nanomolar half maximal inhibitory concentration (IC_50_) values against HDAC1 and HDAC6, along with significant antitumor activity and low toxicity *in vivo* [58]. They used docking programs in Discovery Studio to model these compounds against the structures of HDAC1 and HDAC6 [59,60]. Although a seemingly credible receptor-ligand complex structure was generated, the results indicated that the β-elemene scaffold did not significantly contribute to receptor-ligand binding at the binding site. Besides the docking results, the authors did not present experimental evidence confirming the direct interaction of these two active compounds with HDAC1 and HDAC6. Another potential target of β-elemene is the protein kinase B (Akt)-mechanistic target of rapamycin (mTOR) signaling pathway, essential for regulating cell growth and frequently dysregulated in cancer cells. Considering this, mTOR inhibitors are theoretically capable of suppressing cancer cell growth and proliferation. Xu et al. [61] synthesized 14 β-elemene derivatives, demonstrating that derivatives IIm and IIn inhibited the phosphorylation of Akt, mTOR, and p70S6K in K562 cells at the micromolar level, indicating an inhibition of the Akt-mTOR pathway. However, consistent with previous studies, no receptor-ligand interaction data were presented. Considering that the Akt-mTOR pathway functions downstream of METTL3, it remains possible that these compounds primarily target METTL3, modulating Akt-mTOR signaling. In addition to specific proteins, Zhou et al. [62] recently identified β-elemene as a stabilizing agent for microRNA-145-5p (*miR-145-5p*), binding to its 3′-terminus and enhancing its stability without altering its biosynthetic pathway. This, in turn, inhibits the mitogen-activated protein kinase 3/Nuclear factor kappaB cells (MAPK3/NF-κB) signaling pathway, suppressing the proliferation of non-small cell lung cancer (NSCLC) cells. Unfortunately, this study did not provide atomic-level information that could be used for SBDD. Collectively, as previous target studies have not yielded any significant SBDD-relevant insights for β-elemene, the following discussion will be exclusively dedicated to examining the interaction between β-elemene and its derivatives with METTL3. The current study also explores the feasibility of developing anticancer small molecules using β-elemene as a scaffold structure, specifically targeting METTL3, via CADD techniques.

## 3 Structural perspectives of β-elemene binding

**Fig. 2:**
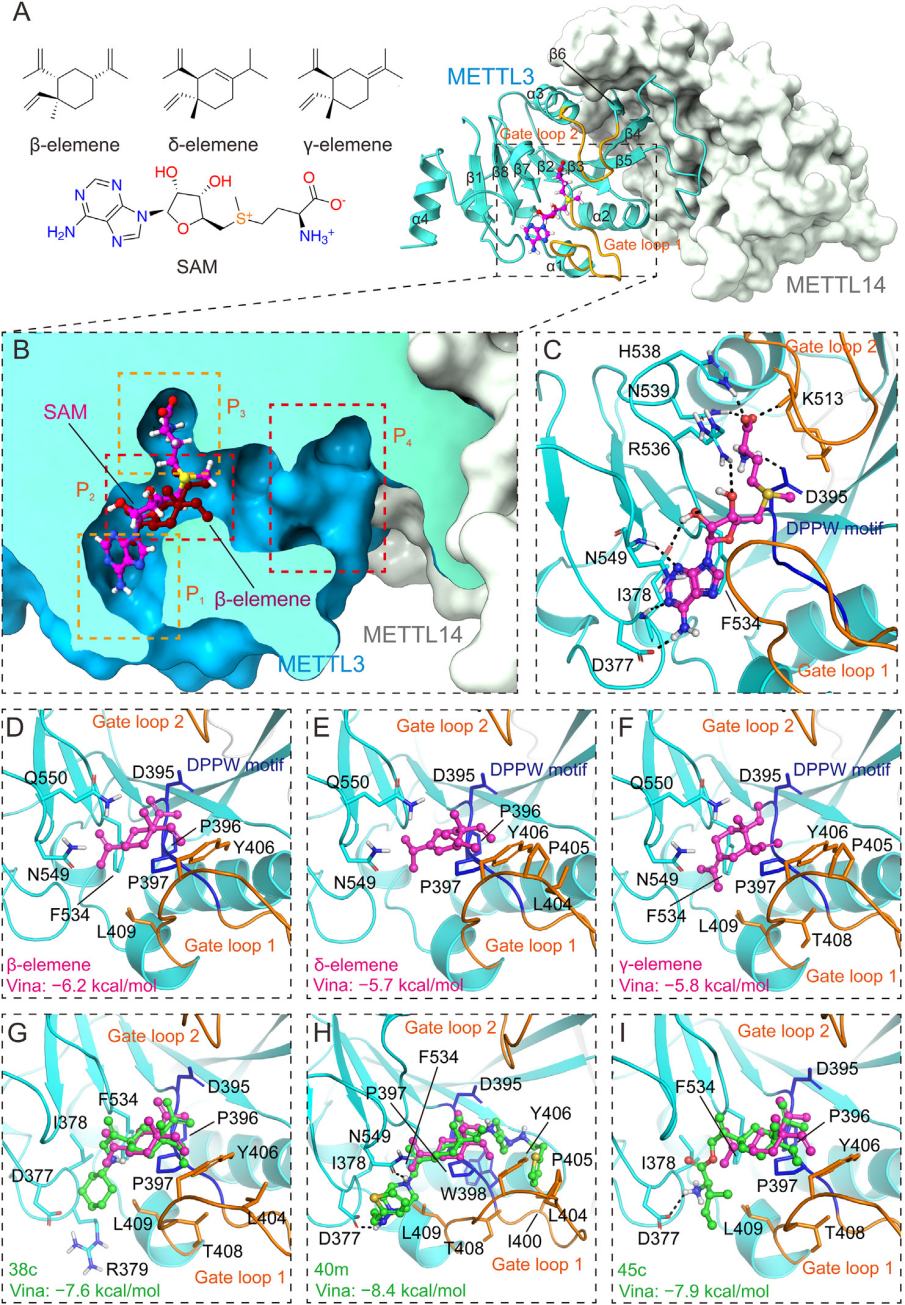
The interaction modes of β-elemene and its isomers and derivatives with methyltransferase-like 3 (METTL3) were predicted by AutoDock Vina, and comparisons were made with the crystal structure of *S*-adenosylmethionine (SAM)-METTL3 (Protein Data Bank (PDB): 5IL1). METTL3 and METTL14) are represented in blue and white, respectively, while the gate loop 1 and gate loop 2, closely related to ligand binding, are shown in orange. (A) The two-dimensional (2D) structures of β-elemene, δ-elemene, γ-elemene and SAM (left) and the global view of METTL14-bound METTL3 (right). (B) A cross-sectional view of the METTL3 binding site when bound to SAM (PDB: 5IL1) is presented, with SAM and β-elemene depicted in different colored ball-stick models. (C) The spatial relationship between SAM and surrounding residues is illustrated, highlighting important interactions. Residues that interact significantly with SAM are shown in stick models, with hydrogen bonds and other polar interactions indicated by black dashed lines, and the DPPW motif is displayed in deep blue. (D–F) The binding conformations of three forms of elemene with METTL3, as predicted by AutoDock Vina. Residues forming significant interactions with the ligands are represented in stick models, and the DPPW motif is highlighted in deep blue: β-elemene with METTL3 (D), δ-elemene with METTL3 (E), and γ-elemene with METTL3 (F). (G–I) The binding conformations of three representative β-elemene derivatives with METTL3, also predicted using AutoDock Vina. To visually compare the position and conformation of the β-elemene scaffold, each figure displays β-elemene and its derivatives in different colored ball-stick models, with residues interacting significantly with the derivatives shown in stick models and polar interactions such as hydrogen bonds indicated by black dashed lines. The DPPW motif is highlighted in deep blue: compounds 38c (G), 40 m (H), and 45c (I) with METTL3.

To conduct SBDD, a comprehensive examination of the ligand-binding site was essential. Using the substrate-bound state of METTL3 with SAM as an example (Protein Data Bank (PDB): 5IL1) (Fig. 2A), the catalytic methyltransferase (MTase) domain of METTL3 was found to comprise four α-helices, eight β-strands, and multiple loops. The SAM binding site was formed by two loops: gate loop 1 (which connected α1 and α2, residues 396–410) and gate loop 2 (which linked α3 and β6, residues 507–515). Together with β1, β7, and β8, these loops constituted the binding pocket [38]. The highly conserved DPPW motif (residues 395–398) is closely related to the catalytic reaction and overlaps extensively with gate loop 1 sequentially. The SAM binding site on METTL3 is characterized by a long and narrow cavity, which Li et al. [55] spatially divided into four distinct regions: P_1_, P_2_, P_3_, and P_4_ (Fig. 2B). The adenosine moiety of SAM was observed to occupy the P_1_ and P_2_ regions, where the adenine formed hydrogen bonds with the backbone atoms of D377 and I378, while the ribose moiety established hydrogen bonds with N549 and R536 (Fig. 2C). The sulfur-containing amino acid moiety penetrates P_3_, forming polar interactions with residues such as K513 on gate loop 2 and D395 in the DPPW motif (Fig. 2C).

Due to the limited studies on the interactions between β-elemene and target proteins, we preliminarily investigated the interaction patterns of β-elemene and its representative derivatives with METTL3 using AutoDock Vina [63,64]. This software, recognized as one of the most widely cited molecular docking tools (with over 20,000 citations) [29], was employed to build upon the work of Liu et al. [14]. Importantly, substantial conformational differences have been observed in gate loop 2 of METTL3 when comparing its ligand-free state (PDB: 5IL0) to its SAM-bound state (PDB: 5IL1) [38].

Based on the current hypothesis, β-elemene, as a potential inhibitor of METTL3, was expected to occupy the SAM binding site in its ligand-free state, thereby preventing SAM binding and then modulating m^6^A modification to exert anticancer effects. To evaluate this possibility, three isomers of elemene (δ, β, and γ) were docked to ligand-free METTL3 (Figs. 2D–F). The results indicated that β-elemene yielded the best docking score (−6.2 kcal/mol) among the three isomers and occupied the P_2_ region of the binding site, showing a high spatial overlap with the ribose moiety of SAM (Fig. 2B). In clinical therapy, as previously noted, β-elemene demonstrated greater effectiveness compared to its δ and γ isomers. This observation corresponded well with the docking results, as reflected in the docking scores provided (Figs. 2D–F).

In the absence of a well-characterized molecular target for β-elemene, substantial advancements have been achieved in synthesizing its derivatives. Different studies have reported the development of halogenated [65,66], amine [61,66,67], ether [67], ester [68], and other structurally complex derivatives of β-elemene [4,69,70], with certain lead compounds revealing markedly enhanced anticancer activity. For this analysis, several active and structurally representative derivatives were selected from the compilation of β-elemene derivatives documented by Bai et al. [56]. AutoDock Vina was used to dock these derivatives to both the ligand-free METTL3 (PDB: 5IL0) and the SAM-bound state of METTL3 (PDB: 5IL1) [38], and the docking results showed that the derivatives were docked much better into the SAM-bound state of METTL3 (Table 1) [56]. This observation was closely associated with the conformation of gate loop 2, which adopted a more open state in the presence of SAM, thereby creating a more accommodating binding pocket. As a result, this particular set of docking results was analyzed in more detail. Importantly, among the 20 docked derivatives, all showed higher docking scores compared to β-elemene, with 10 of them displaying a high degree of spatial overlap with the β-elemene scaffold, as illustrated in Fig. 2D. This alignment was further supported by the root mean square deviation (RMSD) of the cyclohexane moiety, as detailed in Table 1. Meanwhile, the introduced functional groups extended into additional regions of the binding pocket. Among these derivatives, three were identified as the most representative: 38c (amine derivative), 40m (ether derivative), and 45c (ester derivative), as depicted in Figs. 2G–I (compound nomenclature follows the review by Bai et al. [56]). Within the P_2_ region, different residues such as F534, P396, and P397 in the DPPW motif and Y406 in gate loop 1 provide hydrophobic interactions that are crucial for maintaining the occupancy of the β-elemene scaffold in the P_2_ pocket (Figs. 2D and G−I). For the derivatives, the added functional groups extended from the β-elemene scaffold, enabling them to occupy the P1 and P3 pockets and establish a diverse range of interactions with the receptor, as illustrated in Figs. 2G–I. These preliminary docking results indicated that β-elemene and its derivatives were highly likely to bind at the SAM binding site of METTL3.

**Table 1:** The docking results of 20 β-elemene derivatives obtained using AutoDock Vina (all derived from the review by Bai et al. [56], detailed two-dimensional (2D) molecular structures can be found in the original reference).

Derivatives	IC_50_ (μM)	Receptor structure		RMSD of cyclohexane moiety (Å)	Type
		5IL0	5IL1		
38b	0.04	−5.9	−7.0	0.6005	Amine derivatives
38c	3.39	−6.2	−7.6	0.4553	
38f	0.52	−5.9	−7.6	0.5483	
39a	<15	−6.0	−7.0	5.1137	
39b		−5.7	−7.1	5.0869	
39c		−6.2	−7.2	0.5922	
39d		−6.3	−7.3	5.0565	
39e		−6.1	−8.1	0.6558	
40c	3.44	−6.8	−9.0	0.7805	
40 m	<5	−5.3	−8.4	0.6953	
40n		−6.0	−7.8	6.2509	
41c	105.9	−6.1	−6.8	0.2341	Ether derivatives
43	<11	−6.6	−6.6	4.5263	
45a	/	−7.4	−8.9	4.9956	Ester derivatives
45b		−6.0	−7.8	0.8380	
45c		−5.9	−7.9	0.8352	
45d		−6.2	−6.2	5.4163	
45f		−6.8	−8.0	3.3787	
46g	/	−6.7	−8.2	3.0920	
49h	27.5	−7.0	−8.6	3.3767	Amino acid derivatives

/: no data; IC_50_: half maximal inhibitory concentration; RMSD: root mean square deviation.

Current research indicates that the design of METTL3 inhibitors based on SBDD strategies has made some initial progress, but it is still limited within the SAM adenosyl moiety. The SAM analog sinefungin (also named A9145), first isolated by Hamill et al. [71] in 1973, closely resembles SAM in structure, with the main difference being the replacement of the fragment responsible for m^6^A methylation in SAM with different moiety. Bedi et al. [54] established a compound library consisting of 4,000 analogs and derivatives derived from the adenosine moiety of SAM and conducted docking studies on these compounds with METTL3 using the AutoDock program. To confirm that the selected compounds adopted the expected binding modes, 70 compounds were retained from the 120,000 docking-generated conformations based on the criterion that adenine N1 formed a hydrogen bond with I378–NH and adenine N3 with N549–NH. These compounds were selected for activity assays, successfully identifying several inhibitors with credible affinity for METTL3. Moreover, the crystal structures of certain inhibitor-METTL3 complexes were resolved. A comparative analysis between the sinefungin-METTL3 complex structure (PDB: 6Y4G) and the structures of the screened derivatives revealed that these derivatives occupied the binding site as predicted by molecular docking, adhering to the expected binding modes. This SBDD approach depended on previously identified ligand structures and their interaction networks. While it effectively facilitated the rapid identification of lead compounds, its inherent limitations restricted further exploration.

Other binding sites, such as the METTL3-METTL14 interface, also provide the potential for designing small-molecule inhibitors, shifting the mechanism from blocking SAM binding to disrupting the METTL3-METTL14 dimerization. Based on the crystal structure (PDB: 5IL1) of the METTL3-METTL14 dimer in the SAM-bound state, Li et al. [72] designed a short-peptide METTL3-METTL14 disruptor, RSM3, derived from the M3 region of METTL3 (residues 435–447). RSM3 can displace METTL3 by occupying a specific region on METTL14, thereby disrupting the METTL3-METTL14 interaction. RSM3 showed an IC_50_ similar to or even better than that of small-molecule inhibitors (IC_50_ = 4.6 μM against the leukemia cell line CCRF-CEM) and effectively suppressed both cancer cell migration and proliferation. *In vivo* experiments demonstrated that RSM3 significantly reduced tumor size without inducing considerable toxic effects on organs. Based on these findings, an alternative possibility emerged, i.e., β-elemene might have bound to a specific region at the METTL3-METTL14 interface, thereby preventing dimer formation and inhibiting m^6^A methylation levels. This hypothesis required further evaluation via computational drug design techniques and experimental validation in future studies.

## 4 Design strategies for anticancer derivatives of β-elemene via CADD approaches

In the previous section, the flexibility of the receptor protein had not been considered during molecular docking, which might have resulted in outcomes that were not precise and were only appropriate for preliminary assessment. Considering the relatively weak affinity between β-elemene and METTL3, obtaining the complex structure through structural resolution techniques presented a significant challenge. Within this context, computational approaches such as Monte Carlo (MC) sampling or molecular dynamics (MD) simulations have been used to predict the binding conformations of β-elemene with METTL3 while adequately accounting for the flexibility of the binding site [73]. This approach not only provides more accurate binding free energy values but also enables conformational clustering to identify the predominant conformations of the complex. Building upon this foundation for SBDD, virtual screening through molecular docking and AI-driven molecular generation have effectively identified novel β-elemene derivatives showing significant anticancer activity.

### 4.1 Docking-based virtual screening

#### 4.1.1 Implementation basis

When three-dimensional (3D) structures of the target receptor and ligand dataset are available, molecular docking-based virtual screening enables rapid assessment of the binding affinity between multiple compounds and the target. These 3D structures of receptor proteins can be obtained through structural resolving techniques, homology modeling, or AI-based structure prediction [29,74]. In the design of β-elemene derivatives examined in this study, the 3D structures of the relevant METTL3 protein, available in the RCSB database (e.g., PDB IDs: 5IL0, 5IL1, 5IL2, 6Y4G, and others), have been highly suitable for molecular docking-based virtual screening due to their structural integrity and high-resolution data. At that time, various compound libraries were accessible for virtual screening, and they were different in types, scales, and quantities. For example, the structures of all U.S. Food and Drug Administration (FDA)-approved small molecules or drugs can be extensively downloaded from the DrugBank database in multiple formats [75]. Commercial compound libraries historically comprised an extensive collection of structurally diverse molecules, most readily available for direct procurement rather than necessitating custom synthesis, provided they fulfilled the requisite criteria for molecular docking. The ZINC database functioned as a comprehensive repository integrating compounds from various commercial sources, comprising millions of entries [76,77]. Researchers could identify compounds that met specific screening parameters and acquire them in bulk for virtual screening applications. Besides conventional pre-existing compound libraries, Sadybekov et al. [78] introduced a modular dynamic synthesis strategy known as V-SYNTHES, which integrated molecular docking. This approach initially identified the most suitable scaffold-synthon combinations, followed by an iterative synthesis. Researchers implemented this method on actual drug target receptor proteins, substantially increasing the number of screened compounds while significantly enhancing the activity and structural diversity of the resulting lead compounds.

The sampling algorithm and scoring function are the two most critical components in molecular docking programs. The sampling algorithm provides the ligand-receptor binding conformations, while the scoring function assigns a value of predicted binding free energy (or docking score). The effectiveness of these two components directly determines the overall performance of the docking program [79]. Molecular docking programs can be categorized into academic and commercial types. Examples of academic programs include Autodock Vina [63,64], GOLD [80], and UCSF DOCK [81], which are typically developed and maintained by universities or research institutions and are free for academic use. Secondly, commercial docking programs, such as Glide [82,83], MOE Dock [84], and Surflex Dock [85], are often combined with comprehensive CADD software packages. Although commercial software provided a more user-friendly experience, access to its license typically required a fee.

Molecular docking algorithms can be categorized into flexible, semi-flexible, and rigid docking based on the degree of sampling precision. Advancements in computational power had largely rendered rigid docking obsolete, as this approach treated both the receptor and ligand as fixed structures during sampling. In the preliminary stage of virtual screening, semi-flexible docking is usually used to balance accuracy and efficiency, accounting for the flexibility of the ligand only. After ranking and screening compounds in the library based on docking scores, docking evaluation can be performed with higher precision and incorporate the flexibility of site residues to enhance result accuracy [86,87]. When difficulties were encountered in incorporating receptor flexibility during molecular docking, an alternative strategy involved assessing ligand binding affinity across multiple receptor conformations [86]. However, the successful application of this approach necessitated a profound understanding of the system under investigation. After preliminary screening, the molecular mechanics/generalized born surface area (MM/GBSA) method can also be introduced in the further stage, which requires a detailed evaluation. It offers more reliable results than standard molecular docking programs [88].

#### 4.1.2 Cases of docking-based virtual screening targeting METTL3

Molecular docking-based virtual screening has been used for ligand design targeting METTL3. Selberg et al. [89] analyzed the structure of METTL3 in a complex with *S*-adenosyl-l-homocysteine (SAH) (PDB: 5K7W) to examine the interaction patterns between SAM and METTL3. Their findings revealed that the adenine ring of SAM was positioned between F534 and N595, while the ribose moiety formed polar interactions with surrounding residues. Through the application of AutoDock 4.2, a targeted virtual screening was conducted on nitrogen-containing heterocyclic compounds from the DrugBank 4.0 and ZINC databases, resulting in the identification of four candidate compounds. These authors then validated the binding affinities of these four compounds to METTL3 using surface plasmon resonance (SPR) sensorgrams and used MD simulations to explore the interaction patterns of compounds 1 and 4 with the SAM binding site. The results revealed that both compounds showed a high degree of complementarity with the binding site, wherein D395 and K513 engaged in polar interactions with the protonated nitrogen atoms of the ligand's piperidine or piperazine rings. Interestingly, these compounds occupy minimal space within the cavity due to their small molecular volumes. The interaction between their carbonyl oxygen atoms and the sulfur atom of the SAM methylation reaction center may enhance the binding affinity of SAM and potentially lower the energy barrier for substrate RNA methylation reactions. Experimental results suggested that the four compounds act as METTL3-METTL14 agonists; all except compound 4 promoted m^6^A at specific concentrations.

Du et al. [90] aimed to discover anticancer compounds from natural products that regulate m^6^A by binding to METTL3. A library comprising 1,042 commercially available natural products was established for molecular docking studies. AutoDock Vina (v 1.1.2) and the SAM-bound METTL3 structure (PDB: 5IL1) were used to dock and screen the compounds, selecting those with docking scores below −7 kcal/mol (a total of 14 compounds) for further analysis. These selected compounds were procured commercially and assigned to *in vitro* activity experiments.

The results demonstrated that quercetin, scutellarin, and luteolin inhibited m^6^A. Their authors comprehensively investigated MD simulations on quercetin, the most potent inhibitor of METTL3 (docking score: −9.1 kcal/mol, IC_50_ = 2.73 μM). The results showed that the dihydroxyphenyl moiety of quercetin occupies the space between P397, F534, and N549, while its phenolic ring, substituted with hydroxyl groups, fits into the P1 pocket. Multiple hydroxyl groups present in quercetin formed hydrogen bonds with adjacent residues, including N549, C376, and R536. This study provided a remarkable example of identifying METTL3 inhibitors via CADD and offered a valuable foundation for the development of anticancer derivatives derived from β-elemene.

**Fig. 3:**
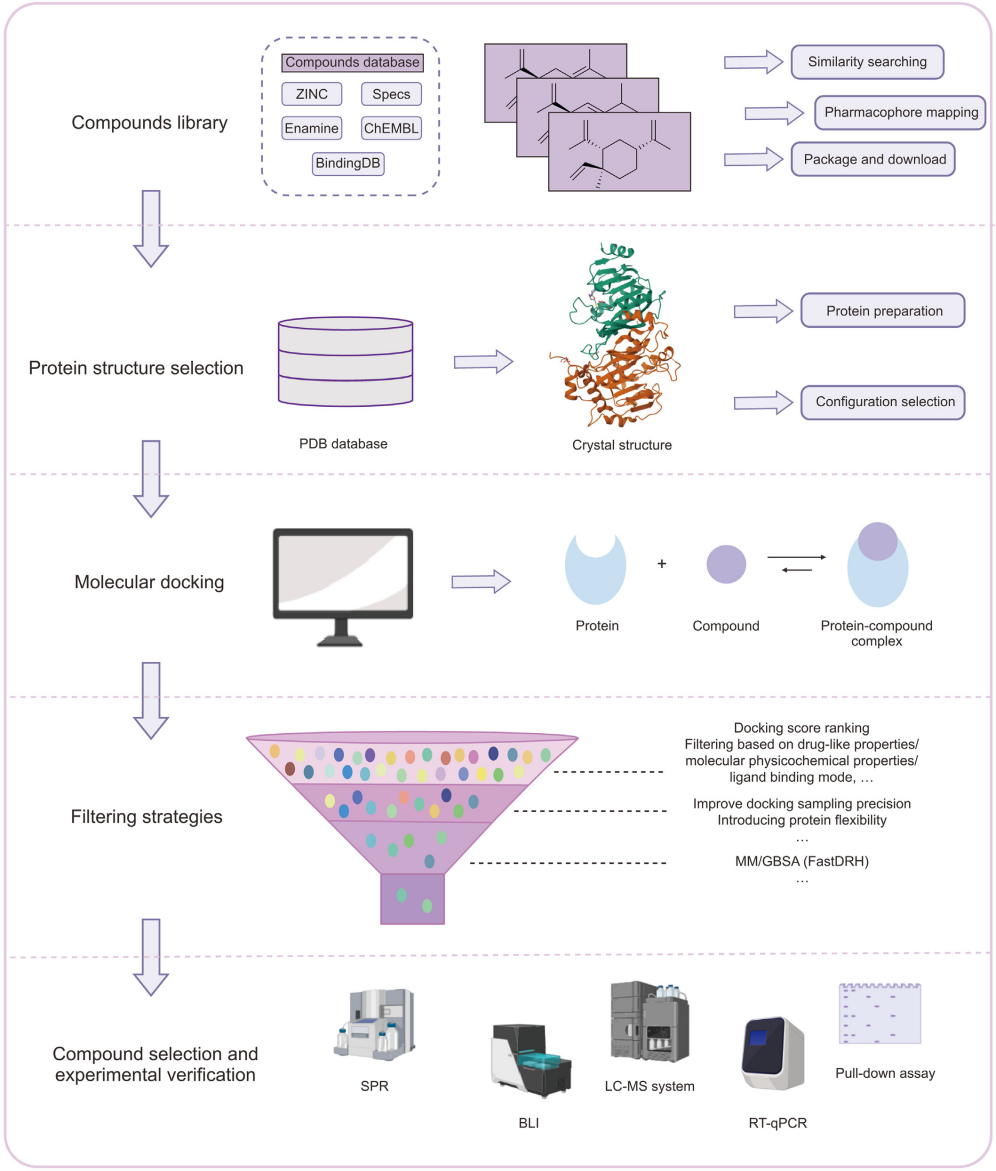
Based on existing virtual screening work targeting methyltransferase-like 3 (METTL3), a refined workflow for obtaining METTL3 inhibitors through docking-based virtual screening is presented. PDB: Protein Data Bank; MM/GBSA: molecular mechanics/generalized born surface area; SPR: surface plasmon resonance; BLI: bio-layer interferometry; LC-MS: liquid chromatography-mass spectrometry; RT-qPCR: reverse transcription quantitative polymerase chain reaction. Created with BioRender.com.

The combination of these two studies with the previous research by Bedi et al. [54] led to the development of a standardized workflow for identifying anticancer lead compounds targeting METTL3 via docking-based virtual screening (Fig. 3). This approach involved selecting appropriate METTL3 crystal structures based on an analysis of ligand-METTL3 interactions, along with the selection of a suitable compound library for docking or the design of a specialized library tailored to specific research objectives. After the initial virtual screening, the selected compounds undergo further filtering. Advanced computational techniques predict their binding affinity to the receptor, facilitating a more precise ranking. Alternatively, compounds may be filtered according to specific customized criteria to enhance selection efficiency. The selected compounds can either be acquired from commercial sources or synthesized through chemical methods before undergoing *in vitro* experiments to evaluate their binding affinity for METTL3 and their inhibitory effectiveness against the protein.

### 4.2 AI-based molecular generation approaches

**Fig. 4:**
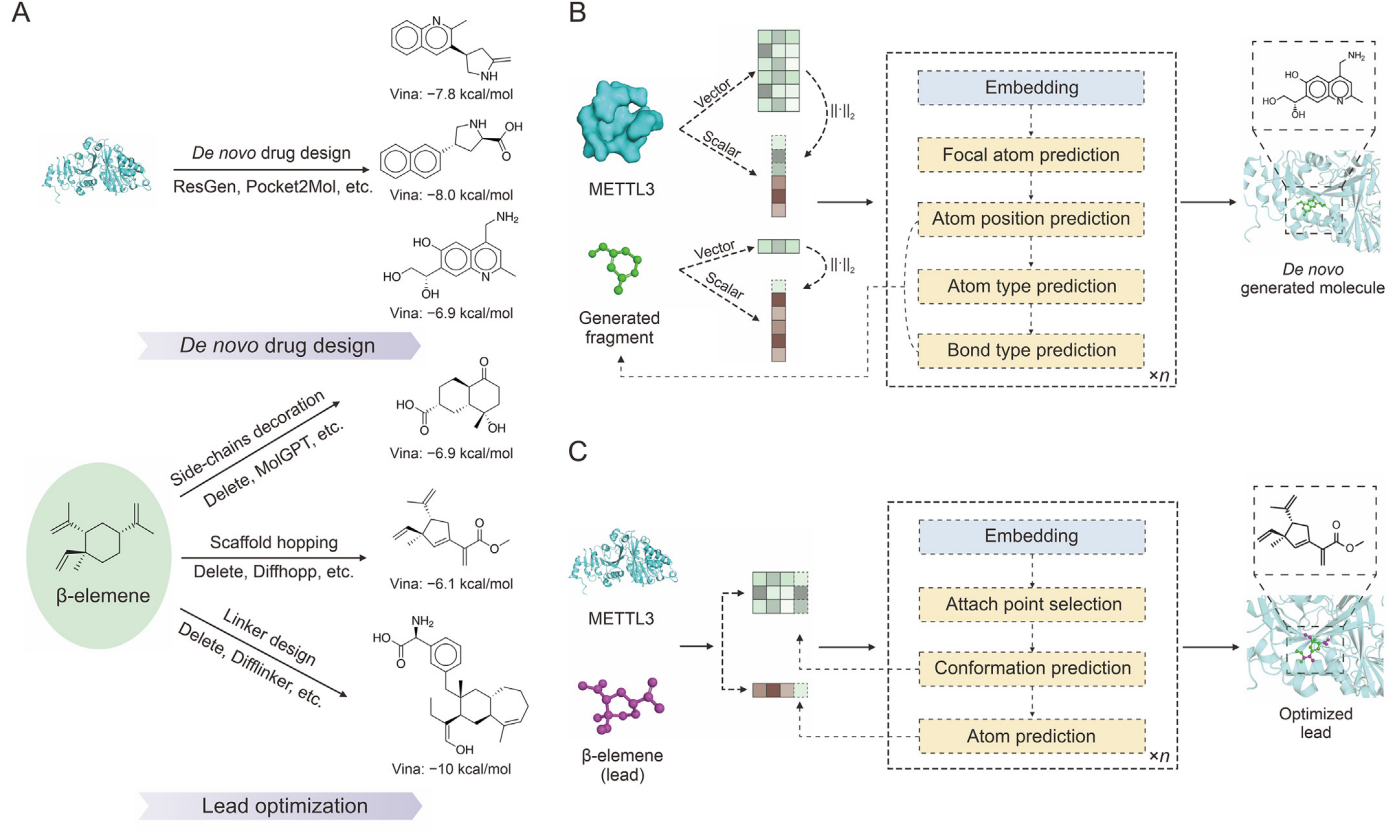
Examples of artificial intelligence (AI)-generated molecules and the workflows of representative models ResGen and Delete. (A) Application cases of molecular generation models in the development of methyltransferase-like 3 (METTL3) small molecule inhibitors based on the β-elemene scaffold. This involves *de novo* molecular generation based on the METTL3 protein and scaffold hopping, linker design, and side-chain decoration based on β-elemene scaffold. (B) The workflow of the ResGen model, where we use green molecular ball-and-stick models to showcase the molecular fragments during the generation process and the final molecular structure. (C) The workflow of the Delete model, where we use purple to represent the retained β-elemene fragments and green to represent the generated molecular fragments.

Molecular generation techniques represent another prominent approach in SBDD, enabling the discovery of drug-like molecules with specific chemical properties. Many studies have demonstrated that rational molecular generation models significantly accelerated the identification of drug candidates [[91], [92], [93]]. Unlike virtual screening techniques, molecular generation was not limited by existing compound databases and theoretically allows for more comprehensive searches of the drug-like space, which had been estimated to range from 10^23^ to 10^60^ scale [94]. The application of molecular generation in practical drug discovery primarily followed two approaches: *de novo* molecular generation and lead compound optimization. ResGen [95] was a representative model for *de novo* molecular generation, while Delete [96] exemplified lead compound optimization. These models were used to conduct a preliminary study into the rational drug design of structure-based drug discovery (SBDD) centered on β-elemene. Fig. 4A presented several candidate compounds identified through these two approaches. A summary of existing *de novo* molecular generation models, along with models designed for lead compound optimization, was provided. This summary categorized the models based on their functional mechanisms, molecular representation methods, and underlying frameworks, as detailed in Table 2 [[95], [96], [97], [98], [99], [100], [101], [102], [103], [104], [105], [106], [107], [108], [109], [110], [111], [112]].

**Table 2:** List of existing artificial intelligence (AI)-based *de novo* drug design models and lead optimization models.

Method	Function	Molecular representation	Model fragment	Refs.
Graph-GMVAE	Scaffold hopping	2D-molecular graph	Graph-based VAE	[101]
DeepHop	Scaffold hopping	SMILES	Transformer	[102]
DiffHopp	Scaffold hopping	3D-molecular graph	Diffusion	[103]
DeLinker	Linker design	2D-molecular graph	VAE	[104]
SyntaLinker	Linker design	SMILES	Transformer	[105]
DEVELOP	Linker design and fragment replacement	3D-molecular graph	VAE	[111]
DiffLinker	Linker design	3D-molecular graph	Diffusion	[107]
3DLinker	Linker design	3D-molecular graph	E(3)-equivariant graph and VAE	[106]
GraphScaffold	Side-chain decoration and fragment replacement	2D-molecular graph	VAE	[108]
MolGPT	Side-chain decoration	SMILES	Transformer-decoder	[109]
DeepFrag	Fragment replacement	Fingerprints	Deep convolutional neural network	[110]
FFLOM	Linker design, side-chain decoration, and fragment replacement	2D-molecular graph	Flow	[112]
Delete	Scaffold hopping, linker design, side-chain decoration, and fragment replacement	3D-molecular graph	Autoregressive model	[96]
GraphBP	*De novo* drug design	3D-molecular graph	Autoregressive model	[97]
Pocket2Mol	*De novo* drug design	3D-molecular graph	Autoregressive model	[98]
ResGen	*De novo* drug design	3D-molecular graph	Hierarchical autoregressive model	[95]
surfGen	*De novo* drug design	3D-molecular graph	Autoregressive model	[99]
FragGen	*De novo* drug design	3D-molecular graph	Fragment-based and autoregressive model	[100]

2D: two-dimensional; VAE: variational autoencoder; SMILES: simplified molecular input line entry system.

#### 4.2.1 De novo molecular generation

*De novo* design of molecules refers to molecular design from scratch based on the physicochemical properties of the binding pocket without relying on an existing small molecule structure. With advancements in technologies such as AlphaFold2 [74], significant progress has been made in leveraging AI methods for 3D molecular design. Early models [113,114] used a geometry-aware framework that considered protein pockets as conditions for ligand-based molecule generation (LBMG), while later LIGAN [115], GraphBP [97], and SBDD [116] allowed for the generation of ligand molecules directly within protein pockets. A geometric neural network was employed in the Pocket2Mol [98] model to enhance the binding affinity of generated small-molecule ligands. The ResGen method further advanced molecular design by capturing higher-level interaction forces through parallel multiscale modeling while also improving computational efficiency. Moreover, ResGen has demonstrated its effectiveness in real-world drug design cases, successfully generating drug-like molecules that bind tightly to protein pockets [95]. The workflow of ResGen is depicted in Fig. 4B, where multiscale modeling involves the abstraction of amino acids into backbone and residue properties. Vector features represented the relative and global positional relationships between amino acids, while scalar features comprised one-hot encoding and dihedral angles of amino acids. For small-molecule ligands, vector features corresponded to the 3D Cartesian coordinates, whereas scalar features included atom types, bonding relationships, and other molecular characteristics. During the generation of ligand molecules, the ResGen model sequentially generates new atoms by predicting subcomponent information for each atom, repeating the process until the molecule reaches a preset maximum molecular weight or the focal atom prediction probability falls below a certain threshold [95].

In *de novo* drug design for small-molecule inhibitors targeting the METTL3 protein based on the β-elemene scaffold, the docking results of β-elemene with METTL3 revealed the binding pocket locations, specifically β-elemene occupying the P2 region of the METTL3 binding site mentioned in Section 3. Next, the ligand-free 3D structure of METTL3 (PDB: 5IL0) can be used as the input file to generate molecules by using models such as ResGen [95], SurfGen [99], and FragGen [100]. A comparative analysis was conducted on all 115 molecules generated using the ResGen model against β-elemene, revealing that 60% of the generated molecules showed better docking scores. Three of the highest-scoring molecules were selected for presentation in Fig. 4A, demonstrating that their Vina docking scores were competitive with the 20 derivatives examined in Section 3. Further modifications and screenings could be performed by medicinal chemists, potentially leading to the identification of novel inhibitors targeting the METTL3 protein.

#### 4.2.2 Lead optimization

Lead optimization entailed introducing structural modifications to existing lead compounds to enhance biological activity, target selectivity, synthetic accessibility, pharmacokinetic properties, and safety. Based on the chemical structure modification strategies, lead optimization can be categorized into four main approaches: scaffold hopping, linker design, side-chain decoration, and fragment replacement [117]. The lead optimization of the β-elemene scaffold focused on improving its physicochemical properties, including water solubility and molecular stability, while preserving its affinity for target proteins. Further, efforts were made to explore and identify structurally novel compounds with potential bioactivity.

##### 4.2.2.1 Scaffold hopping

Scaffold hopping represented a widely used approach in lead optimization, enabling the identification of novel compounds that maintained similar pharmacological properties to the original scaffold while possessing a structurally distinct chemical backbone. This approach is widely used in drug discovery, not only to optimize the molecular properties of existing scaffolds but also to identify biologically active molecules that can bypass the patent barriers of existing drug-like compounds [118,119]. Graph-GMVAE [101] was the first deep generative model specifically developed for scaffold hopping. This model replaced the conventional single Gaussian distribution in the latent space with a multivariate Gaussian distribution, where the distance between two points within the latent space reflected the similarity between different chemical scaffolds, thereby facilitating controlled scaffold hopping. DeepHop [102] redefined scaffold hopping as a language translation problem, using the simplified molecular input line entry system (SMILES) of the original lead compound as input. Moreover, protein sequence information was incorporated via a Transformer model, enabling the translation of molecules into structurally similar compounds in 3D space while maintaining distinct 2D scaffolds. DiffHopp [103] advanced this approach by developing a 3D diffusion generative model that conditioned the scaffold hopping on the 3D structure of the protein pocket. This design explicitly accounted for geometry-aware interactions between the ligand and the protein, ensuring a more precise optimization process. Furthermore, for molecules containing linkers within their scaffold, deep generative methods originally designed for linker optimization, such as Delinker [104] and SyntaLinker [105], were also used to facilitate simple scaffold hopping. In the development of small molecule METTL3 inhibitors based on the β-elemene scaffold, the SMILES structure of β-elemene and the METTL3 protein sequence can be input into DeepHop to generate new molecules through scaffold hopping. Alternatively, DiffHopp can be used to obtain 3D molecular structures and binding conformation of the newly generated molecules within the METTL3 protein pocket, using the 3D structural data of both β-elemene and the METTL3 protein as inputs.

##### 4.2.2.2 Linker design

The purpose of linker design is to connect the identified functional fragments, using the entropy reduction effect to lower the overall free energy of binding between the molecule and the protein pocket, thereby stabilizing the interaction. A well-designed linker allows the connected molecule to retain the original binding mode of the lead fragments with the protein pocket [120,121]. DeLinker [104] was the first molecular generation model specifically developed for linker design. SyntaLinker [105] used Transformer models to analyze the syntactic patterns within SMILES, thereby identifying the rules for the connection of functional fragments in existing compound structures. However, these models were restricted to generating 2D linker structures and did not account for the geometric interactions within the protein pocket. To overcome this limitation, 3DLinker [106] incorporated equivariant neural networks to determine the coordinates of the linking atoms at each iteration step, enabling a more precise consideration of the geometric conformation of the linker and its interactions with the protein pocket. DiffLinker [107] allows the design of linkers for an arbitrary number of fragments, rather than being limited to connecting just two active fragments as in previous methods. In the development of METTL3 inhibitors, although β-elemene itself cannot be further fragmented for linker redesign, the active portions of β-elemene and the amino acid segments of SAM that extend into the P_3_ region of the binding pocket to form polar interactions with residues such as D395 in the DPPW motif, can be viewed as two active fragments of a new lead compound. The lead compound was optimized to enable its occupation of the P_2_ and P_3_ regions of the METTL3 binding pocket.

##### 4.2.2.3 Side-chain decoration

The successful development of a drug often involves multiple candidates based on the same core scaffold. This development paradigm typically begins by identifying a favorable scaffold that binds to the target, followed by the addition of various modifying groups at different positions on the scaffold to enhance the binding affinity or drug-like properties of the chemical derivatives [122]. GraphScaffold [108] represented the first deep learning-based approach for side-chain decoration. This method employed a sequential decision-making strategy, wherein new atoms and bonds were incrementally added to the compound scaffold in an autoregressive manner to facilitate side-chain modifications. Inspired by generative pre-trained (GPT) models, MolGPT [109] used a masked self-attention mechanism to capture the semantic relationships within SMILES. Furthermore, MolGPT enabled limited molecular generation by allowing the input of specific target molecular properties, such as logP, synthetic accessibility (SA) score, topological polar surface area (TPSA), and the quantitative estimate of drug-likeness (QED), as predefined constraints. In the development of small-molecule METTL3 inhibitors based on the β-elemene scaffold, β-elemene can serve as a starting point, using models like GraphScaffold to explore derivatives that share the same core scaffold. The objective was for the side chains of these derivatives to extend into the P_1_ and P_3_ regions of the binding pocket, leading to candidate compounds with enhanced binding affinity.

##### 4.2.2.4 Fragment replacement

In the early stages of lead compound discovery, fragment optimization was used to fill the cavities in the protein pocket, thereby enhancing the binding affinity of the lead compounds. Compared to side-chain decoration, fragment replacement is often associated with the concept of pharmacophores, and the optimized groups are typically larger, frequently containing ring structures. DeepFrag [110] approached the fragment optimization problem by converting it into a classification task through the development of a fragment library, whereas DEVELOP [111] addressed the constraints of fragment libraries by incorporating a generative model, variational autoencoder (VAE). Next, FFLOM [112] improved the quality of fragment replacement by implementing flow models that facilitated reversible transformations between the model's latent space and the chemical space. In the development of small-molecule METTL3 inhibitors based on the β-elemene scaffold, the relatively simple structure of β-elemene was not suitable for this method; future developments can involve modifications based on existing small-molecule METTL3 inhibitors, such as sinefungin.

Most models mentioned above can only perform molecular generation for one or two sub-tasks within lead optimization. However, the Delete [96] model unified the majority of lead compound optimization tasks by implementing a standardized mask strategy, offering a comprehensive solution. Fig. 4C depicts the workflow of the Delete model. In Delete, the first step in generating new compounds involves selecting attach points from existing lead compounds, followed by developing the relative positions and coordinates of new atoms, and finally predicting the types of atoms and their bonding relationships with the existing atoms. This process is continuously repeated until the molecule is fully generated. Here, the Delete model was used to optimize lead compounds derived from the β-elemene scaffold. During the scaffold hopping, only a portion of the side chains of β-elemene was preserved, and the retained side-chain fragments, along with the ligand-free 3D structure of METTL3 (PDB: 5IL0), were used as input files. This strategy successfully generated 102 novel molecules. The docking scores of the newly generated compounds were evaluated using AutoDock Vina, with nearly all molecules (97%) showing improved scores compared to the β-elemene scaffold. For linker design, the β-elemene scaffold was retained and combined with amino acid fragments from the SAM structure as two active fragments to be connected. This resulted in 105 compounds with significantly improved binding affinities, with the average docking score of the top 10 molecules assessed by Autodock Vina being −13.4 kcal/mol. The substantial improvement in binding affinity may stem from the entropy reduction effect achieved through effective linker design. In side-chain decoration, only the six-membered ring at the center of the β-elemene scaffold was retained as the input, resulting in 130 new structures, most of which showed better docking scores (70%). Here, one molecule from each of the three lead optimization methods was selected for presentation in Fig. 4A.

## 5 Conclusion

β-elemene, an anticancer natural product extracted from TCM, holds unique advantages and broad potential in cancer treatment. The application of SBDD could inject new vigor into the development of β-elemene scaffold anticancer agents. A clear and specific target is the foundation of SBDD. Here, a comprehensive review of existing studies on the molecular mechanisms of β-elemene was conducted, focusing on its interactions with the METTL3. This METTL3 was most likely the primary target protein responsible for the anticancer effects of β-elemene, while other potential targets, such as HDAC, had not yet been validated. Molecular docking results indicated that β-elemene and its derivatives occupied the P_2_ binding site of the METTL3, primarily interacting with key residues, i.e., F534, P396, P397 in the DPPW motif, and Y406 in gate loop 1. Virtual screening studies targeting METTL3 were briefly reviewed, along with the application of AI-based molecular generation models in *de novo* drug design and lead optimization. This study provided preliminary insights into SBDD for the development of β-elemene derivatives. Thus, the AI-driven molecular generation models showed promising results in the *de novo* molecule generation based on METTL3 and the lead optimization as per the β-elemene scaffold. Most candidate molecules obtained improved Vina docking scores with METTL3 comparing with β-elemene while presenting innovative molecular structures. However, further experimental validation remained essential to determine whether METTL3 was the target protein of β-elemene. Computationally predicted molecular structures generated by molecular generation models also required experimental evidence to substantiate their reliability. Moreover, despite the exciting advancements demonstrated by many AI models in drug discovery, structure-based molecular generation still requires further exploration. While generative models can produce molecules with potential biological activity, they often serve merely as references and inspiration for medicinal chemists, requiring further modification and screening. Extensive collaboration between medicinal chemists and algorithmic models could result in the development of other β-elemene-based anticancer drugs, providing significant advancements and potential therapeutic outcomes for cancer patients.

## CRediT authorship contribution statement

**Haiyi Chen:** Writing – review & editing, Writing – original draft, Visualization, Validation, Supervision, Software, Project administration, Methodology, Investigation, Formal analysis, Data curation, Conceptualization. **Yuntao Yu:** Writing – review & editing, Writing – original draft, Visualization, Validation, Methodology, Investigation, Formal analysis, Data curation. **Chenghong Hu:** Writing – review & editing, Visualization, Validation, Supervision, Methodology, Investigation. **Lehuang Zhou:** Validation, Formal analysis, Data curation. **Zhe Wang:** Software, Resources, Methodology. **Odin Zhang:** Writing – review & editing, Supervision. **Yi Wang:** Supervision, Funding acquisition. **Tian Xie:** Writing – review & editing, Validation, Supervision, Resources, Project administration, Investigation, Conceptualization.

## Declaration of competing interest

The authors declare that there are no conflicts of interest.
